# Sonochemical Deposition of Gentamicin Nanoparticles at the PCV Tracheostomy Tube Surface Limiting Bacterial Biofilm Formation

**DOI:** 10.3390/ma16103765

**Published:** 2023-05-16

**Authors:** Łukasz Ścibik, Dorota Ochońska, Monika Gołda-Cępa, Konrad Kwiecień, Elżbieta Pamuła, Andrzej Kotarba, Monika Brzychczy-Włoch

**Affiliations:** 1 Department of Molecular Medical Microbiology, Chair of Microbiology, Faculty of Medicine, Jagiellonian University Medical College, 18 Czysta Street, 31-121 Kraków, Poland; 2 Faculty of Chemistry, Jagiellonian University, 2 Gronostajowa Street, 30-387 Kraków, Poland; mm-golda@uj.edu.pl (M.G.-C.); kotarba@chemia.uj.edu.pl (A.K.); 3 Department of Otolaryngology and Oncological Surgery of the Head and Neck, 5th Military Hospital with Polyclinic in Krakow, 1-3 Wrocławska Street, 30-901 Kraków, Poland; 4 Department of Biomaterials and Composites, Faculty of Materials Science and Ceramics, AGH University of Science and Technology, Al. Mickiewicza 30, 30-059 Kraków, Poland

**Keywords:** tracheostomy tube, gentamicin nanoparticles, surface functionalization, bacterial adhesion

## Abstract

Background: The use of nanotechnology in the production of medical equipment has opened new possibilities to fight bacterial biofilm developing on their surfaces, which can cause infectious complications. In this study, we decided to use gentamicin nanoparticles. An ultrasonic technique was used for their synthesis and immediate deposition onto the surface of tracheostomy tubes, and their effect on bacterial biofilm formation was evaluated. Methods: Polyvinyl chloride was functionalized using oxygen plasma followed by sonochemical formation and the embedment of gentamicin nanoparticles. The resulting surfaces were characterized with the use of AFM, WCA, NTA, FTIR and evaluated for cytotoxicity with the use of A549 cell line and for bacterial adhesion using reference strains of *S. aureus* (ATCC^®^ 25923™) and *E. coli* (ATCC^®^ 25922™). Results: The use of gentamicin nanoparticles significantly reduced the adhesion of bacterial colonies on the surface of the tracheostomy tube for *S. aureus* from 6 × 10^5^ CFU/mL to 5 × 10^3^ CFU/mL and for *E. coli* from 1.655 × 10^5^ CFU/mL to 2 × 10^1^ CFU/mL, and the functionalized surfaces did not show a cytotoxic effect on A549 cells (ATTC CCL 185). Conclusions: The use of gentamicin nanoparticles on the polyvinyl chloride surface may be an additional supporting method for patients after tracheostomy in order to prevent the colonization of the biomaterial by potentially pathogenic microorganisms.

## 1. Introduction

Tracheotomy is the most often performed procedure in patients in cases of upper airway obstruction or prolonged mechanical ventilation. Of all patients hospitalized in intensive care units, 12–20% require this treatment [1,2]. Tracheotomy became an object of interest of scientists in March 2020, when WHO announced the COVID-19 pandemic, and gained importance among patients requiring long-term ventilator therapy [3]. This procedure can be performed surgically or percutaneously using dedicated tools. Both techniques are characterized by a minimal risk of complications and can be used in hospitalized patients after considering the contraindications to a given technique [4].

Each medical device that encounters the patient’s tissues is exposed to potential contact with pathogenic microorganisms. The tracheostomy tube, due to its application and contact with three different environments—the patient’s tissue, air, and secretions—is particularly exposed to the rapid development of bacterial biofilm, and is thus a source of nosocomial infections [5]. Among the complications of the tracheostomy procedure are local and generalized infections, most often infections associated with long-term mechanical ventilation [6]. For this reason, it is important to develop alternative effective methods of combating emerging biofilm on the surface of medical devices.

In the times of modern medicine, the use of various polymers for dedicated implant applications has become a permanent part of the standards of patient treatment. Polymeric biomaterials are used in each field of medicine to produce external equipment (syringes, catheters, bags, etc.) as well as implants permanently placed inside the human body (vascular stents, breast implants, hip prostheses, etc.) [7]. The dynamic development of nanomaterials and nanopolymers has further consolidated the position of biomaterials in the field of medical science. They have been used, for example, in drug-releasing systems (including drugs used in cancer therapies), anticorrosion coatings of metal implants, or antibacterial membranes used in wound treatment [8]. Among the most popular polymers, polyvinyl chloride is widely used. About 32% of medical devices in hospitals are made of this material [9]. That is the reason why PVC is still extensively investigated to improve its properties and therapeutic functions.

The formation of a biofilm on the surface of biomaterials, including PVC, is one of the biggest problems with recurrent infections. The term bacterial biofilm refers to clusters of bacterial colonies attached to surfaces or to each other and surrounded by a self-created bacterial matrix. The matrix is a mixture of various biochemical substances such as proteins, polysaccharides, and genetic material derived from bacterial colonies. These molecules form a natural protective barrier, the crossing of which is a major challenge in the treatment of biofilm-associated infections. Bacterial colonies suspended in a biofilm are characterized by increased resistance to antibiotics, becoming invisible to the host’s immune system and showing greater tolerance to an anaerobic environment with reduced access to nutrients. All these features mean that infections associated with biofilms are usually chronic, and the only effective method to stop them is the removal of the biomaterial and surgical intervention for the inflamed tissue [9,10].

Gentamicin belongs to the aminoglycoside group of antibiotics isolated from Micromonospora purpurea. It was discovered in 1963 and introduced for medical use in 1971. Its activity is associated with binding to the 30S subunit of the ribosome, which disrupts the translation process in the bacterial cell. Thanks to its antibacterial properties, it is applied in the treatment of bacterial infections in orthopedics, cardiology, pulmonology, neurology, and laryngology. Unfortunately, it also has several side effects: nephrotoxicity, ototoxicity, or neurotoxicity. Hence, it is important to limit its systemic impact, e.g., by local delivery and action [11]. Its use in the form of nanoparticles acting directly at the place of implantation of the tracheostomy tube may be an alternative to its oral administration, which can substantially reduce its side effects.

In this study, it was decided to synthesize and immediately deposit gentamicin nanoparticles onto the PVC surface using the ultrasonic technique. The use of ultrasounds to produce nanoparticles of controlled size and morphology has proven to be an effective method for various chemical substances including bioactive molecules. When solutions are exposed to ultrasounds (in the range of 20 kHz–1 MHz) the phenomenon of acoustic cavitation leads to bubbles’ formation and growth. If the molecules of interest are present in the solution, some population of them is located at the bubble–liquid interface. On reaching a critical volume, the bubbles implode and the dissolved substance located at the interface forms nanoparticles. The molecular mechanism of this phenomenon was recently described elsewhere [12].

Nowadays, nanomedicine has become one of the branches of dynamic research and is mainly focused on the development of new methods of preventing and treating diseases. The use of nanomedicines allows the drug doses used in prevention to be controlled compared to their classic versions [13]. One of the promising research directions in nanomedicine is the use of targeted therapies and drug release in cancer treatment regimens [14]. The main advantage of drugs used in nanomedicine is the possibility of using lower doses of antibiotics to obtain the same therapeutic effects. This reduces the risk of developing multidrug resistant strains. Nanomedicine can not only reduce the dose of the antibiotic, but also increase its activity. One excellent example of increasing activity while reducing the drug dose is the use of encapsulated vancomycin in hybrid magnetic nanoparticles conjugated to a cell-penetrating peptide to fight against *S. aureus* and *E. coli* strains [15].

The main objective of the work was to functionalize the surface of typical tracheostomy tubes made of polyvinyl chloride (PVC) using oxygen plasma with the sonochemical incorporation of gentamicin nanoparticles. Such a functionalization allows for local antibiotic delivery and the prevention of infections, the most common complication after tracheostomy. The materials were biologically tested in terms of their cytotoxicity (A549 ATCC^®^ CCL185) and risk of bacterial adhesion (*Staphylococcus aureus* ATCC^®^ 25923™, *Escherichia coli* ATCC^®^ 25922™).

## 2. Materials and Methods

### 2.1. Biomaterial Characteristics

In this study, polyvinyl chloride biomaterials supplied by the producer of tracheostomy tubes (SUMI, Sulejówek, Poland) were used in the form of cut discs 15 mm in diameter and 2 mm thick, supplied in packages of 50 pieces in sterile conditions.

### 2.2. Oxygen Plasma Modification

Oxygen plasma (FEMTO system, Diener Electronics, Ebhausen, Germany) was used to generate surface functional groups containing oxygen atoms. Optimization of the plasma modification parameters of polyvinyl chloride samples was carried out using variable oxygen pressure, generator power, and treatment time. The effect of surface functionalization was carried out by monitoring the changes in water contact angle. The measurements were performed using a goniometer (Surftens Universal Instrument, OEG GmbH, Hessisch Oldendorf, Germany). The images were analyzed using the Surftens 4.3 software (OEG GmbH). For each sample, 5 measurements (droplet volume 2 μL) were taken and the obtained values were averaged.

### 2.3. Preparation of Gentamicin Nanoparticles and Their Instant Deposition on PVC Surface

To deposit the antibiotic on the PVC surface, nanoparticles were produced from an aqueous solution of pure gentamicin by sonication, after prior treatment of the surface with oxygen plasma.

The synthesis of gentamicin nanoparticles and their deposition was performed in a single-stage process on a PVC disc with a diameter of 15 mm, modified with oxygen plasma, supplied by the manufacturer of tracheostomy tubes. A solution of gentamicin sulfate in distilled water at a concentration of 50 mg/mL was used for the experiments. The deposition of nanoparticles was carried out using a homogenizer (Vibracell CV18, Sonics & Materials Inc., Newtown, CT, USA). Process parameters: frequency, 20 kHz; amplitude, 30%; time, 6 min. To increase the efficiency of the synthesis of nanoparticles, 0.5 mL of ethanol (Avantor Performance Materials, Gliwice, Poland) was added to the prepared 3 mL solution of gentamicin. After sonication, the material was dried on adsorptive nonwoven cloth to remove excess solution from the surface [16].

#### Nanoparticle Tracking Analyses (NTA)

The size distribution of the sonochemically produced gentamicin nanoparticles was determined using an LM10 Nanosight instrument (Malvern Instruments Ltd., Malvern, UK) equipped with an sCMOS camera (Hamamatsu Photonics, Hamamatsu, Japan) using a non-blue 450 nm laser. Data analysis was performed using NTA software (3.1 Build 3.1.45). Measurement parameters: camera level, 15; shutter, 1206; gain, 366. A single measurement consisted of three 30 s video clips recorded at 25 frames/s.

### 2.4. Characterization of Functionalized Biomaterial

#### 2.4.1. Fourier Transform Infrared Microscopy (FTIR)

To investigate the presence of gentamicin nanoparticles on the PVC surface, FTIR analysis was performed. Characteristic bindings for gentamicin were sought. The analysis of the surface was performed using a Nicolet 6700 Thermo Scientific in the range of 4000−650 cm^−1^, and the resultant spectra were averaged across 64 scans. Spectra in the range of 850–2000 cm^−1^ were used for the analysis, in which the most important changes were observed.

#### 2.4.2. Analysis of Drug Loading on the Biomaterial Surface

For the quantitative analysis of the gentamicin loading, the kinetic of drug release was monitored for parent and modified PVC surfaces. The release profile was studied using the OPA-based method. Each of the prepared discs was placed in a vial with 20 mL of PBS buffer for 5 days and continuously mixed at 100 rpm at 37 °C. To compare the results, an experiment was performed on polyvinyl chloride discs with gentamycin nanoparticles before and after oxygen plasma treatment. At fixed time intervals (2 h), 2 mL of PBS were taken from the vials, the collected liquid was placed in 2 mL Eppendorf tubes, and the vials were replenished with 2 mL of fresh PBS solution. The amount of drug released was quantified using an OPA assay, as described before [17]. Then, 30 mg of OPA (orto-phthaldialdehyde, Sigma-Aldrich, St. Louis, MO, USA) was dissolved in 0.5 mL of methanol (Sigma-Aldrich) and 0.1 mL of mercaptoethanol (Sigma-Aldrich), and the solution was added to 50 mL of borate buffer (pH = 10.4). The reagent thus obtained was mixed with 50 uL of the collected sample at a fixed time interval in a 1:1 volume ratio and incubated in the dark for 10 min in a black 96-well plate. After that, fluorescence was measured in an excitation wavelength of 340–310 nm and an emission wavelength of 460 nm (FLU-Ostar Omega, BMG Labtech, Ortenberg, Germany).

#### 2.4.3. Atomic Force Microscopy (AFM)

The surfaces of PVC samples before and after the plasma treatment were investigated with the use of NanoWizard^®^ 4XP (Brucker, Billerica, MA, USA) equipped with SCANASYST-AIR (Brucker) probes. The images (20 × 20 µm, 256 × 256 px) were collected with the set of the following parameters: setpoint, 9.252 nN; Z length, 650 nm; Z speed, 79.11 um/s; px time, 20.54 ms. The analysis was performed with JPK Data Processing software (ver. 7.0.153).

### 2.5. Microbiological Tests

#### 2.5.1. Bacterial Strains

Two reference (indicator) strains were used in the study, including a representative of the Gram-positive bacteria *Staphylococcus aureus* ATCC25923 and a representative of the Gram-negative bacteria *Escherichia coli* ATCC25922. The strains were from the American Type Culture Collection, ATCC. The selection of strains was based on a previous study of clinical material from patients with a tracheostomy [18].

#### 2.5.2. Quantification of Bacterial Adhesion on the Surface of Polyvinyl Chloride

From the reference bacterial strains of *Staphylococcus aureus* and *Escherichia coli* after an 18 h incubation, 0.5 McF suspensions were obtained in Tryptic Soy Broth (TSB). The functionalized and control discs were placed in sterile polystyrene 24-well microtiter plates (Costar^®^ Corning) and 1 mL of bacterial inoculum was added. The plates were incubated for 2 h at 37 °C, and the attached bacterial colonies were detached from the biomaterial. The resulting bacterial suspension was plated at 100 μL on Tryptic Soy Agar (TSA, Becton Dickinson) growth medium in serial ten-fold dilutions. After 24 h of incubation at 37 °C, the result was read in CFU/mL.

#### 2.5.3. Evaluation of the Cytotoxicity of Gentamicin (GMNPs) on the A549 Cell Line

The adenocarcinomic human alveolar basal epithelial cell line A549 was used to test the cytotoxicity of gentamicin. Cells were grown in DMEM medium with -L-glutamine supplemented with 10% FBS and 1% ZellShield (MB Minerva Biolabs, Berlin, Germany) at 5% CO_2_ and 37 °C with L-alanyl-L-glutamine. Cells were harvested with 0.5% trypsin, suspended in 5 mL DMEM. The cells contained in 1 mL of the suspension were counted and diluted to give 1 × 10^4^ cells per sample. The experiment was carried out in 96-well plates. Then, 200 µL of solution with a decreasing gentamicin concentration (10–0 mg) was added to the cell cultures. The MTT test was used to determine the metabolic activity of the cells. The results were read on an Infinite^®^ M Nano (TECAN) microplate reader at 563 nm after 1 and 24 h of incubation [19].

#### 2.5.4. Evaluation of the Effect of Polyvinyl Chloride Surface on the A549 Cell Line Using the LIVE/DEAD Method

The cell lines were prepared as in Section 2.5.3. PVC discs before and after functionalization were placed in 24-well plates, filled with cell suspension, and incubated for 24 h. Then, double staining was applied to the tested surfaces using the LIVE/DEAD^®^ kit (Live/Dead Cell Double Staining Kit, Molecular Probes, Eugene, OR, USA). The use of the double staining method using the LIVE/DEAD kit allows for an easy and quick determination of cell viability in a population based on the integrity of the cell membrane. Fluorescein diacetate, due to its low molecular weight, can penetrate inside the cell with preserved cytoplasmic membranes. The second dye, propidium iodide, PI, having a high molecular weight, penetrates only into cells with damage in the cytoplasmic membrane. The dyes used in a fluorescence microscope BX63 (Olympus, Tokyo, Japan) divide the cells into live (green fluorescence) and dead (red fluorescence) [20].

### 2.6. Statistic

All experiments were performed in triplicate on separate samples. The data were treated using the Statistica software 13.1 (TIBCO Software Inc., Palo Alto, CA, USA), applying parametric *t*-student tests, and a *p*-value < 0.05 was considered statistically significant. The error presented in the figures represents the standard deviation of the data.

## 3. Results

### 3.1. Optimization of Oxygen Plasma Parameters

**Figure 1:**
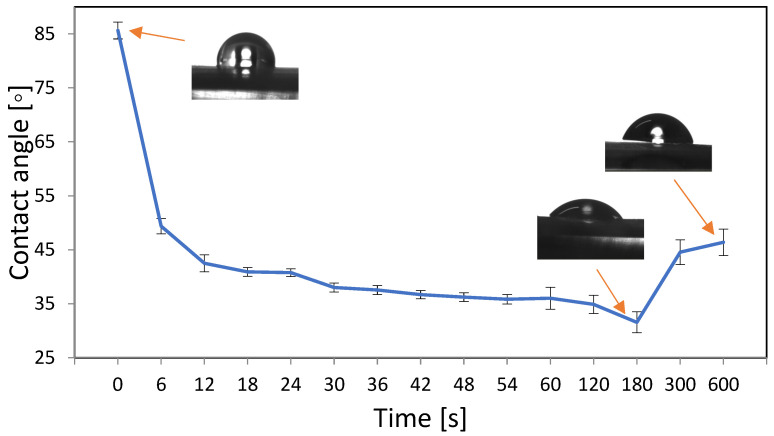
Changes in water contact angle as a function of oxygen plasma treatment time 0–600 s, 50 W, 0.2 mbar. The lowest contact angle was obtained for samples treated for 180 s.

The systematic water contact angle measurements were taken for PVC material treated with oxygen plasma with the use of the following sets of parameters: 50 W, 0.2 mbar, 6–600 s (Figure 1). The contact angle for PVC without surface modification by plasma was 86° ± 2°. The smallest contact angle of the surface was obtained after 180 s of plasma treatment with a power of 50 W and a pressure of 0.2 mbar of oxygen, and was equal to 32° ± 2°. From the profile in Figure 1, the contact angle decreased most rapidly for the first 12 s of plasma exposure, and then the curve flattened out and decreased after 180 s before increasing again.

Based on the results, it was found that the most optimal parameters for modifying the PVC surface with oxygen plasma were 180 s, 50 W, and 0.2 mbar.

The formation of reactive functional groups containing oxygen atoms increased the hydrophilicity of the surface. It is important that this process takes place without damaging the bulk properties of the surface, which were tested by the AFM observation described in the next section.

### 3.2. AFM Imaging for Polymer Topography

**Figure 2:**
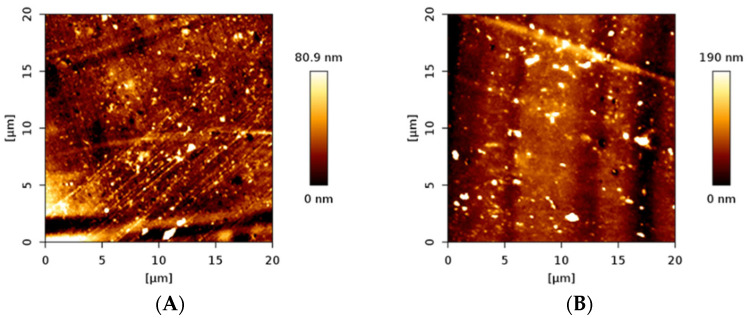
The AFM images of unmodified (**A**) and plasma (50 W, 0.2 mbar of O^2^ and 180 s)-modified (**B**) PVC surfaces.

The topography of the parent and oxygen-plasma-modified PVC surface was characterized by AFM. The comparison is shown in Figure 2. The surfaces represent typical polymeric materials with characteristic groves that originated from the production process. Additionally, some nanometric debris can be noted. In general, the same topography was essentially observed for both samples, indicating that the plasma treatment did not change it at the microscale. It is also worth mentioning that the changes in contact angle resulted from the polar functional groups generated by the applied mild plasma treatment, and not from the topographical changes. Such effects have been previously reported for other polymeric materials [21].

### 3.3. Analysis of Gentamicin Nanoparticles’ Size

**Figure 3:**
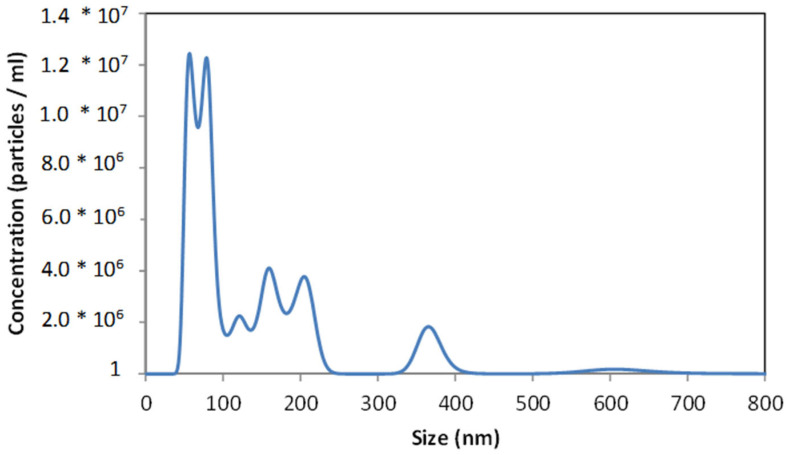
NTA imaging. Size distribution of gentamicin nanoparticles obtained sonochemically.

Figure 3 shows the size distribution of sonochemically prepared gentamicin nanoparticles. Several maxima in the range of 50–350 nm can be distinguished. The first ones, at 50 and 75 nm, were sharp and high, while the other maxima were wider and lower. For the design and manufacture of drug delivery systems, the size distribution of nanoparticles is crucial, and the use of the NTA technique provides this information in detail. The analysis took 90 s, which was enough time to observe the nanoparticles’ agglomeration, so several successive peaks with lower maxima can be observed. The maxima representing individual nanoparticles were those with the highest intensity, i.e., 50 and 75 nm, and the numerous maxima at ~125, 150, 225, and 375 nm were multiplications associated with the aggregation process over time. The intensity of the last maxima increased with time.

### 3.4. Fourier-Transform Infrared Spectroscopy (FTIR)

**Figure 4:**
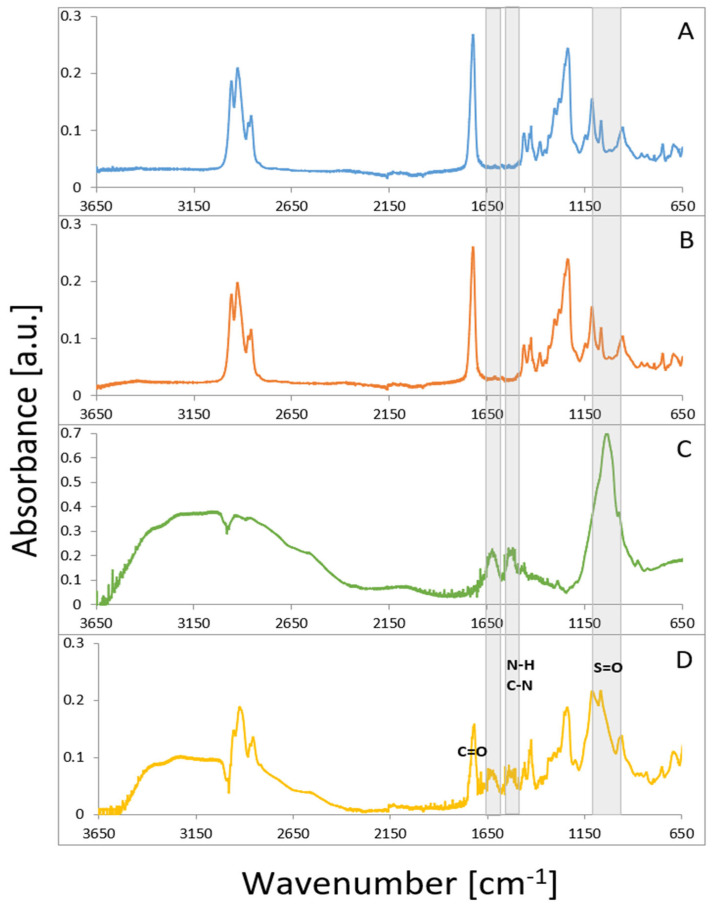
FTIR spectra of PVC (**A**), oxygen-plasma-modified PVC (**B**), gentamycin sulfate (**C**) and after sonochemical incorporation of gentamicin nanoparticles onto the PVC surface (**D**). Gray shadowing indicates characteristic regions for gentamicin sulfate.

The analysis of the IR spectra showed no effect of the oxygen plasma on the PVC structure (Figure 4). The spectrum for the gentamicin-loaded polymeric sample showed characteristic absorption bands at 1616, with 1558 cm^−1^ belonging to the amide I (C=O stretching mode), and amide II (N–H bending mode, and C–N stretching mode) present in gentamicin. The peak located at 1030 cm^−1^ was associated with the HSO4^−^ group characteristic for the sulfate form of the drug [22]. In the 3000–3600 cm^−1^ region, a broad maximum, observed for gentamicin functionalized PVC, was associated with hydrogen-bonded OH groups, indicating their involvement in the surface anchoring of the drug. The presence of these peaks confirms that gentamicin nanoparticles were successfully embedded into the PVC surface. No structural changes in the drug molecule or biomaterial bulk properties were observed after the functionalization (plasma and ultrasound treatments). These observations are essential for preserving the bioactivity of the antibiotic and providing a therapeutic function for the PVC material.

### 3.5. Drug Loading on the Surface of PVC

**Figure 5:**
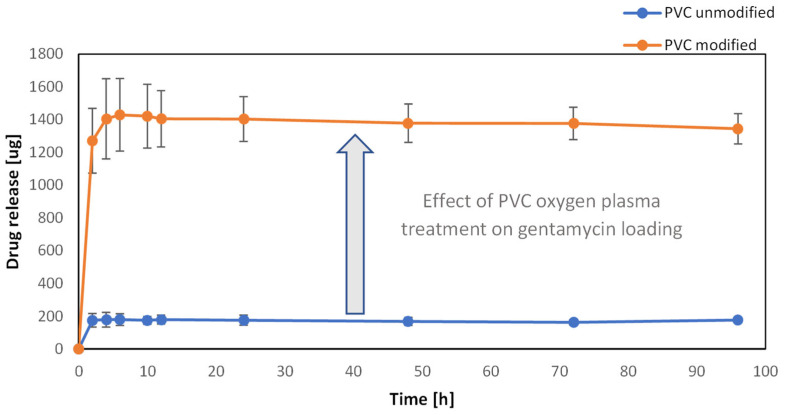
Kinetic curves of gentamicin release from the surface of PVC, functionalized (orange graph) and not functionalized (blue graph) with oxygen plasma (180 s, 50 W, 0.2 mbar).

The gentamicin release kinetics method was used to quantify the loading efficiency of gentamicin on the PVC surface and the effect of its plasma functionalization. As the concentration in the substrate solution was too high to assess the residual concentration of gentamicin after the sonification, the drug loading was estimated to be at the obtained concentration of the release study, when the amount of gentamicin in the buffer solution had not been rising anymore. All of the drug has been released from the surface of modified and unmodified samples in 8 h and 2 h, respectively. The comparison between the samples before and after oxygen plasma treatment is shown in Figure 5. The release profiles show an eight-fold increase in the amount of gentamicin for the sample with surface oxygen groups (orange curve) compared to the untreated sample (blue curve). It can thus be concluded that the generated surface groups led to the formation of adsorption sites for the effective non-covalent bonding of gentamicin molecules on the PVC surface. Such a spectacular effect of increasing the drug loading on the PVC has important practical implications. The ~1.5 mg of gentamicin on the polymeric biomaterial surface of ~2 cm^2^ was within the therapeutic window for in-site release, e.g., gentamicin-loaded sponges, Garamycin Sponge (EUSA Pharma, Hertfordshire, UK) [23].

### 3.6. Adhesion of Bacteria on the Surface of the Biomaterial

**Figure 6:**
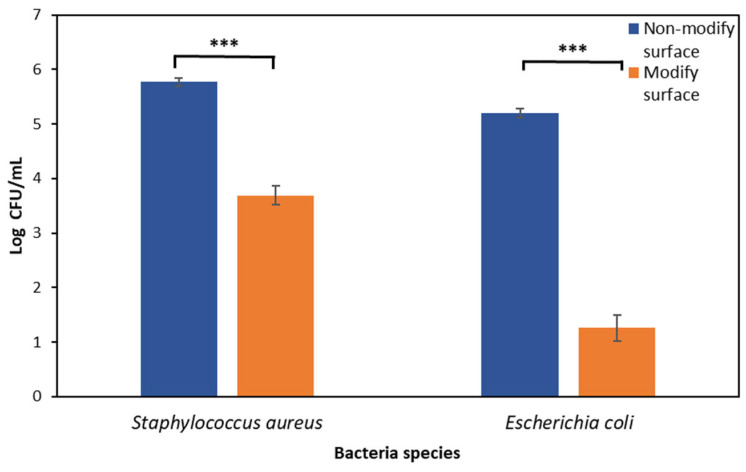
Logarithm of the CFU after incubation of the bacterial suspension at a concentration of 0.5 McF on the PVC surface (orange graph) and on the PVC surface with gentamicin nanoparticles (blue graph) for 2 h for *S. aureus* and *E. coli* (*** results statistically significant between the indicated surfaces, *p* < 0.001, Student’s *t*-test).

The study assessed the effect of the surface of polyvinyl chloride with gentamicin nanoparticles on the adhesion of bacteria (test group) in relation to the control group, which was a sterile, non-functionalized disc (Figure 6). The average CFU/mL from the tested samples of the tested bacterial strains of *S. aureus* and *E. coli* was determined. The antibacterial property was shown to decrease the number of bacteria from 5.77 log CFU/mL to 3.69 log CFU/mL of PVC modified with gentamicin nanoparticles (GMNPs) against *S. aureus* ATCC25923 (*p* < 0.05). The antibacterial property was demonstrated by a decrease in the number of bacteria from 5.2 log CFU/mL to 1.26 log CFU/mL PVC modified with gentamicin nanoparticles (GMNPs) against *E. coli* ATCC25922 (*p* < 0.05). A statistically significant inhibitory effect of nanoparticles on adhesion to polyvinyl chloride was confirmed.

### 3.7. Cytotoxic Effect of Gentamicin Nanoparticles on A549 Cell Line

**Figure 7:**
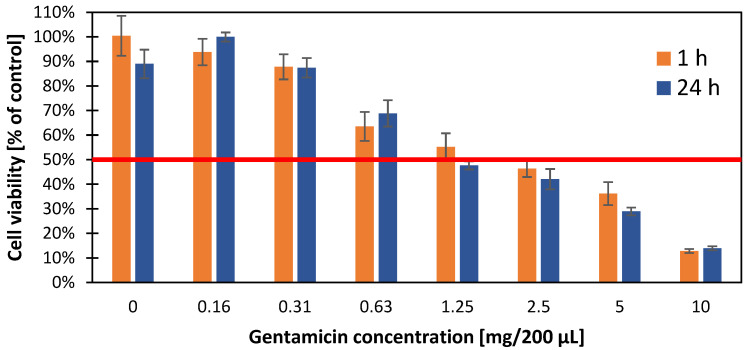
Evaluation of the cytotoxicity (relative to the control) of the gentamicin concentration against the A549 cell line after 1 h (orange) and 24 h (blue) incubation using the MTT colorimetric assay. The results were read using a TECAN reader at a wavelength of 563 nm. A 50% inhibition of growth of the cell population tested is indicated by a horizontal red line.

To investigate the effect of gentamicin nanoparticles on the A549 cell line and their survival, the MTT colorimetric test was performed to determine half of the maximum inhibitory concentration after exposure to gentamicin (Figure 7). IC-50 was evaluated in 2-time intervals (1 and 24 h), setting its value at the level of 1.25 mg/200 μL (6.25 mg/1 mL). The greatest decrease in viable cells was observed after exceeding the concentration of 0.31 mg/200 μL (1.55 mg/1 mL) from 95% to 60% at a concentration of 0.63 mg/200 μL (3.15 mg/1 mL). No increased cytotoxicity was observed with the time of exposure to the antibiotic.

### 3.8. Cytotoxicity of the Tested Surfaces on the A549 Cell Line

**Figure 8:**
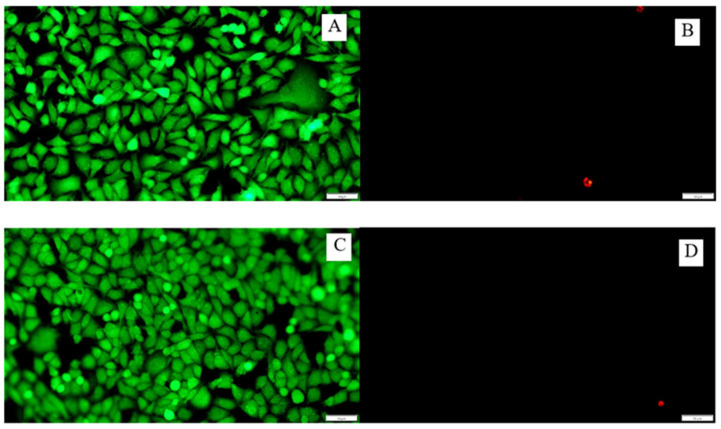
Cells of the A549 line seen under a fluorescence microscope after 24 h incubation on PVC discs without modification (**A**,**B**) and with gentamicin nanoparticles (**C**,**D**). The LIVE/DEAD method was used to image the cells. Live cells were stained green, dead cells stained red. White bars correspond to 50 μm.

In the cytotoxicity evaluation study on the A549 cell line, no significant effect of functionalized surfaces (Figure 8A,B) was observed in relation to the control group (Figure 8C,D) on cell death, which proves its biocompatibility. The dyes used in a LIVE/DEAD method divided the cells under a fluorescence microscope into live (green fluorescence) and dead (red fluorescence), which can be seen in the figure below.

## 4. Discussion

This paper highlights the possibility of the sonochemical incorporation of antibiotics on the surface of the tracheostomy tube to slow down bacterial adhesion. Similar studies on gentamicin and its bactericidal properties were carried out on other materials in different forms or using different technologies for its placement on the surface, as well.

The choice of an antibiotic for the functionalization of the polyvinyl chloride surface was dictated by scientific reports [18,24]. Ravenendra et al., in their research on bacterial biofilm, pointed to the greatest effectiveness of gentamicin and ciprofloxacin in the fight against bacterial biofilm on the surface of the tracheostomy tube [24]. In our own research on bacterial biofilm, we also noted the high effectiveness of gentamicin against bacterial strains isolated from patients with a tracheostomy [18]. The use of gentamicin for the treatment of infections of the etiology of Gram-negative aerobic bacteria was a breakthrough in the prevention of biofilm formation on their basis, including for those associated with the presence of *Pseudomonas aeruginosa*. For over 50 years of its history, infections caused by multidrug-resistant bacteria have been successfully treated, e.g., *Klebsiella pneumoniae*, *Escherichia coli*, *Serratia marcescens*, *Staphylococcus* spp. Initially used parenterally since the 1990s, gentamicin has been used for topical treatment in the form of a collagen sponge, ointment, inhalation, and others in the rapidly developing branch of nanomedicine [25].

In most cases, however, polymer surfaces are chemically inert and devoid of reactive groups that can bind to other molecules on their surface. To increase the adhesive properties of the poly (vinyl chloride) surface, oxygen plasma treatment was used, thanks to which functional groups containing oxygen were generated. Such polar groups make the surface hydrophilic and form active centers for gentamicin molecule adsorption. The drugs can later be delivered to a specific environment because of the action of stimuli-responsive polymers [26]. In addition, the -COOH and -OH groups, due to the accumulated local charges, may negatively affect the adhesion of bacterial cells, thus preventing infection of the functionalized surface [27].

The effect of plasma treatment was monitored using the value of the contact angle of the surface. The preferential contact angle for biomaterials used in medicine should be less than 65 degrees [28]. This is important for biocompatibility with the tissues surrounding the biomaterial. The moderate hydrophilic properties of the biomaterial provide optimal conditions for cell proliferation and differentiation [29]. In addition, the hydrophilic character of the material reduces the adhesion of bacteria to the surface. In this work, the optimal parameters at which the smallest contact angle of the surface (32° ± 2°) was obtained were reached after 180 s of exposure to 50 W plasma and 0.2 mbar oxygen pressure. Comparable results were obtained by Balazs [30]. In his experiment, after 120 s of oxygen plasma operation, the percentage of concentration of oxygen atoms on the PVC surface was the highest.

Such a strategy of surface functionalization with gentamicin on another popular medical polymer—polyethylene—has been reported elsewhere [31]. In another study, Bo-Yu Peng emphasized that the process of PVC depolymerization in the presence of intestinal bacteria is inhibited by using gentamicin. This additionally emphasizes the importance of using this antibiotic in our study on tracheostomy tubes [32].

Gentamicin nanoparticles were created and incorporated into the biomaterial using a homogenizer and ultrasonic technology. The sonochemical process can be used for the formation of nanoparticles of bioactive substances but also, which is of particular interest, the ultrasonic waves facilitate the direct embedment of the nanoparticles into the polymeric surfaces. This is due to ultrasonic energy access, which facilitates the loosening of the polymeric material structure. Such a sonochemical method was successfully employed for the fabrication and embedment of gentamicin nanoparticles into the surface of parylene C used for metal implant coating [16]. Here, we applied the same approach and showed that the method can be used for the functionalization of the PVC material used for the production of tracheostomy tubes.

We did not find gentamicin macromolecules in the tested solution, and the largest nanoparticles had a size of ~350 nm. The largest number of nanoparticles was obtained for a size of 50–75 nm. In a study by Kamal et al. [33], using the sol-gel method, they obtained nanoparticles with a size of 68 nm and confirmed their higher efficiency compared to standard gentamicin. The multiplicity of nanoparticles is of great importance in phagocytosis and pinocytosis. The larger the nanoparticle, the more difficult it is for it to get inside the cell using the above mechanisms. Hence, the smaller the size of the nanoparticle, the greater its biological activity [34]. Since drug nanoparticles are directly deposited on the polymeric surface during the sonochemical process, their stability in the solutions were of secondary importance for the proposed approach and we did not investigate it in detail.

In our study, the loading of gentamicin was performed in two steps. In the first stage, as a result of surface treatment with oxygen plasma, covalent bonds of groups -CO, COOH, OH with the PVC surface were formed. In the second stage, non-covalent bonds were formed between the functionalized surface and gentamicin (through the use of a large number of weak interactions such as electrostatic, ionic, van der Walls and hydrophobic interactions, adsorption, and hydrogen bonds) [25].

According to the analysis of bacterial adhesion on the PVC surface, it was possible to reduce the population of selected strains statistically significantly. Reducing the number of multidrug-resistant bacteria reduces the risk of developing systemic infections, including pneumonia, due to prolonged mechanical ventilation.

The therapeutic dose of gentamicin administered parenterally is 3–6 mg/kg of body weight per day [35]. Due to the narrow therapeutic range, patients should be monitored, especially those with impaired renal function. Gentamicin penetrates well into soft tissues (the highest concentration in the kidneys was 25–100 times higher than in serum), but in the bronchial secretion it is only 25%, which limits its use in patients with a tracheostomy [36]. Therefore, in the case of indications for its administration, it seems important to deliver it topically. The incorporation of gentamicin nanoparticles onto the surface of the tracheostomy tube enables the use of smaller doses of the antibiotic to fight infections resulting from the presence of an artificial opening in the patient’s neck. The tests performed confirmed the possibility of using the sonochemical method to functionalize the surface of the polyvinyl chloride material.

An important aspect is the cytotoxicity of gentamicin, especially in terms of its nephrotoxicity, neurotoxicity, and ototoxicity. Research by Kovacik et al. [37] on VERO cell lines (kidney epithelial cells extracted from African green monkeys) showed a decrease in cell survival and the inhibition of mitochondrial activity at gentamicin concentrations above 2 mg/mL. Similarly, research by Rathbone et al. [38], who evaluated the effect of various antibiotics, including gentamicin, on osteoblast viability, showed a decrease in cell count (>75%) with higher doses of gentamicin (>2 mg/mL). Our studies showed a decrease in the cell viability of the A549 line at concentrations >1.5 mg/mL; however, they seem to be more resistant to gentamicin, as at concentrations of 6.25 mg/mL 50% of the cells remained viable.

## 5. Conclusions

The incorporation of nanoparticles onto the surface of tracheostomy tubes can significantly reduce the adhesion of bacteria to their surface and, thus, inhibit the formation of a bacterial biofilm. These studies provide a good example of how to effectively fight microorganisms while reducing the dose of gentamicin through targeted on-site delivery. They also illustrate the efficacy of combined oxygen plasma (generation of surface polar groups) and sonochemical (embedment of gentamicin nanoparticles) functionalization of the PVC tracheostomy tubes to improve their therapeutic and bactericidal performance.

## Data Availability

The data presented in this study are available on request from the corresponding author.

## References

[1] Abril M.K.M., Berkowitz D.M., Chen Y.M., Waller L.A., Martin G.S.M., Kempker J.A.M. (2021). The Epidemiology of Adult Tracheostomy in the United States 2002–2017: A Serial Cross-Sectional Study. Crit. Care Explor..

[2] Lewith H., Athanassoglou V. (2019). Update on management of tracheostomy. BJA Educ..

[3] Ferro A., Kotecha S., Auzinger G., Yeung E., Fan K. (2021). Systematic review and meta-analysis of tracheostomy outcomes in COVID-19 patients. Br. J. Oral Maxillofac. Surg..

[4] Klotz R., Probst P., Deininger M., Klaiber U., Grummich K., Diener M.K., Weigand M.A., Büchler M.W., Knebel P. (2018). Percutaneous versus surgical strategy for tracheostomy: A systematic review and me-ta-analysis of perioperative and postoperative complications. Langenbecks Arch. Surg..

[5] Ochońska D., Ścibik Ł., Brzychczy-Włoch M. (2021). Biofilm Formation of Clinical *Klebsiella pneumoniae* Strains Isolated from Tracheostomy Tubes and Their Association with Antimicrobial Resistance, Virulence and Genetic Diversity. Pathogens.

[6] Delaney A., Bagshaw S.M., Nalos M. (2006). Percutaneous dilatational tracheostomy versus surgical tracheostomy in critically ill patients: A systematic review and meta-analysis. Crit. Care.

[7] Ratner B.D., Zhang G. (2020). A History of Biomaterials. Biomater. Sci..

[8] Bayda S., Adeel M., Tuccinardi T., Cordani M., Rizzolio F. (2019). The History of Nanoscience and Nanotechnology: From Chemical-Physical Applications to Nanomedicine. Molecules.

[9] Lebeaux D., Ghigo J.M., Beloin C. (2014). Biofilm-related infections: Bridging the gap between clinical management and fundamental aspects of recalcitrance toward antibiotics. Microbiol. Mol. Biol. Rev..

[10] Vestby L.K., Grønseth T., Simm R., Nesse L.L. (2020). Bacterial Biofilm and Its Role in the Pathogenesis of Disease. Antibiotics.

[11] Chytrosz-Wrobel P., Golda-Cepa M., Kubisiak P., Kulig W., Cwiklik L., Kotarba A. (2023). Sonochemical Formation of Fluorouracil Nanoparticles: Toward Controlled Drug Delivery from Polymeric Surfaces. ACS Appl. Nano Mater..

[12] Chaves B.J., Tadi P. (2022). Gentamicin. StatPearls.

[13] Yang Z., Kang S.G., Zhou R. (2014). Nanomedicine: De novo design of nanodrugs. Nanoscale.

[14] Taheri-Ledari R., Zhang W., Radmanesh M., Mirmohammadi S.S., Maleki A., Cathcart N., Kitaev V. (2022). Multi-Stimuli Nanocomposite Therapeutic: Docetaxel Targeted Delivery and Synergies in Treatment of Human Breast Cancer Tumor. Small.

[15] Zhang W., Taheri-Ledari R., Hajizadeh Z., Zolfaghari E., Ahghari M.R., Maleki A., Hamblin M.R., Tian Y. (2020). Enhanced activity of vancomycin by encapsulation in hybrid magnetic nanoparticles conjugated to a cell-penetrating peptide. Nanoscale.

[16] Golda-Cepa M., Chytrosz P., Chorylek A., Kotarba A. (2018). One-step sonochemical fabrication and embedding of gentamicin nanoparticles into parylene C implant coating: Towards controlled drug delivery. Nanomedicine.

[17] Kwiecień K., Pudełko I., Knap K., Reczyńska-Kolman K., Krok-Borkowicz M., Ochońska D., Brzychczy-Włoch M., Pamuła E. (2022). Insight in Superiority of the Hydrophobized Gentamycin in Terms of Antibiotics Delivery to Bone Tissue. Int. J. Mol. Sci..

[18] Ścibik Ł., Ochońska D., Gołda-Cępa M., Brzychczy-Włoch M., Kotarba A. (2022). Microbiological analysis of tracheostomy tube biofilms and antibiotic resistance profiles of potentially pathogenic microorganisms. Otolaryngol. Pol..

[19] Śliwka L., Wiktorska K., Suchocki P., Milczarek M., Mielczarek S., Lubelska K., Cierpiał T., Łyżwa P., Kiełbasiński P., Jaromin A. (2016). The Comparison of MTT and CVS Assays for the Assessment of Anticancer Agent Interactions. PLoS ONE.

[20] (2015). ibidi GmbH, Version 2.0 (Online). https://ibidi.com/img/cms/support/AN/AN33_Live_Dead_staining_with_FDA_and_PI.pdf.

[21] Golda-Cepa M., Brzychczy-Wloch M., Engvall K., Aminlashgari N., Hakkarainen M., Kotarba A. (2015). Microbiological investigations of oxygen plasma treated parylene C surfaces for metal implant coating. Mater. Sci. Eng. C.

[22] Cavalu S., Roiu G., Pop O., Heredea D.A.P., Costea T.O., Costea C.F. (2021). Nano-Scale Modifications of Amniotic Membrane Induced by UV and Antibiotic Treatment: Histological, AFM and FTIR Spectroscopy Evidence. Materials.

[23] Maczynska B., Secewicz A., Smutnicka D., Szymczyk-Ziółkowska P., Dudek-Wicher R., Junka A., Bartoszewicz M. (2019). In vitro efficacy of gentamicin released from collagen sponge in eradication of bacterial biofilm preformed on hydroxyapatite surface. PLoS ONE.

[24] Raveendra N., Rathnakara S.H., Haswani N., Subramaniam V. (2021). Bacterial Biofilms on Tracheostomy Tubes. Indian J. Otolaryngol. Head Neck Surg..

[25] Chen C., Chen Y., Wu P., Chen B. (2014). Update on new medicinal applications of gentamicin: Evidence-based review. J. Formos. Med. Assoc..

[26] Akdogan E., Sirin H.T. (2021). Plasma surface modification strategies for the preparation of antibacterial biomaterials: A review of the recent literature. Mater. Sci. Eng. C.

[27] Rezaei F., Shokri B., Sharifian M. (2016). Atmospheric-pressure DBD plasma-assisted surface modification of polymethyl methac-rylate: A study on cell growth/proliferation and antibacterial properties. Appl. Surf. Sci..

[28] Vogler E.A. (1999). Water and the Acute Biological Response to Surfaces. J. Biomater. Sci. Polym. Ed..

[29] Shafei S., Foroughi J., Chen Z., Wong C.S., Naebe M. (2017). Short Oxygen Plasma Treatment Leading to Long-Term Hydrophilicity of Conductive PCL-PPy Nanofiber Scaffolds. Polymers.

[30] Balazs D., Favez D., Chevolot Y., Xanthopoulos N., Granges C., Aronsson B.-O., Sidouni F., Descouts P., Mathieu H.J. (2001). Surface modification of PVC endotracheal tubes: Oxygen plasma treatment and aging effects. Eur. Cells Mater..

[31] Kumar R.M., Gupta P., Sharma S.K., Mittal A., Shekhar M., Kumar V., Kumar B.M., Roy P., Lahiri D. (2017). Sustained drug release from surface modified UHMWPE for acetabular cup lining in total hip implant. Mater. Sci. Eng. C.

[32] Peng B.-Y., Chen Z., Chen J., Yu H., Zhou X., Criddle C.S., Wu W.-M., Zhang Y. (2020). Biodegradation of Polyvinyl Chloride (PVC) in *Tenebrio molitor* (Coleoptera: Tenebrionidae) larvae. Environ. Int..

[33] Kamal R., Kahdhum Q.A., Mohammed A.I., Essa A.J., Elhamid M.A.A., Mohamad E.A. (2021). Evaluation the Biological Activity of Nano-Gentamicin Prepared and Char-acterization of Nanoparticles. Iraqi J. Ind. Res..

[34] Barar J. (2015). Bioimpacts of nanoparticle size: Why it matters?. Bioimpacts.

[35] Beraud G., Moal G., Elsendoorn A., Tattevin P., Godet C., Alfandari S., Couet W., Roblot P., Roblot F. (2012). A survey on the use of gentamicin in infective endocarditis. Eur. J. Clin. Microbiol. Infect. Dis..

[36] Pennington J.E., Reynolds H.Y. (1973). Concentrations of gentamicin and carbenicillin in bronchial secretions. J. Infect. Dis..

[37] Kovacik A., Tvrda E., Fulopova D., Cupka P., Kovacikova E., Zbynovska K., Massanyi P. (2017). In Vitro Assessment of Gentamicin Cytotoxicity on the Selected Mammalian Cell Line (Vero cells). Adv. Res. Life Sci..

[38] Rathbone C.R., Cross J.D., Brown K.V., Murray C.K., Wenke J.C. (2011). Effect of various concentrations of antibiotics on osteogenic cell viability and activity. J. Orthop. Res..

